# Prenatal diagnosis and genetic counseling of an inherited Xq24q25 deletion associated with normal phenotype

**DOI:** 10.1186/s13039-022-00626-w

**Published:** 2022-11-03

**Authors:** Yaqing Zhou, Mingxi Zhang, Yanmin Zhu, Qi Zhao

**Affiliations:** 1 Reproductive Center Laboratory, Ninghai County Maternal and Child Health Hospital, Ningbo, Zhejiang People’s Republic of China; 2 Department of Internal Medicine, Wuhan Hospital of Traditional Chinese Medicine, Wuhan, Hubei People’s Republic of China; 3 Department of Treatment and Prevention, Wuhan Hospital of Traditional Chinese Medicine, Wuhan, Hubei People’s Republic of China; 4 Department of Clinical Laboratory, Dongsheng Area People’s Hospital, Ordos, Inner Mongolia People’s Republic of China

**Keywords:** Chromosomal microarray analysis (CMA), Noninvasive prenatal testing (NIPT), Chromosomal deletions/duplications, Prenatal diagnosis

## Abstract

**Background:**

Copy number variants (CNVs) are an important source of normal and pathogenic genome variations. CNVs identified in prenatal cases need careful considerations and correct interpretation if those are harmless or harmful variants from the norm.

**Case presentation:**

A 28-year-old, gravida 1, para 0, woman underwent amniocentesis at 17 weeks of gestation because the noninvasive prenatal testing (NIPT) results revealed a 9.8 Mb deletion from Xq24 to Xq25. GTG-banding karyotype analysis was performed on cultured amniocytes. Chromosomal microarray analysis (CMA) on uncultured amniocytes was performed.

**Results:**

Chromosomal GTG-banding of the cultured amniocytes revealed a karyotype of 46,XX. CMA detected a 9.5-Mb chromosomal deletion in the region of Xq24q25 (arr[GRCh37] Xq24q25(118,975,436_128,444,692) × 1).

**Conclusion:**

The present report highlights that an integration of prenatal ultrasound, NIPT, karyotype analysis, CMA and genetic counseling is helpful for the prenatal diagnosis of chromosomal deletions/duplications.

## Introduction

Besides whole chromosome gains or losses, microdeletions and microduplications are in the focus of prenatal diagnostics [1]. Nowadays especially noninvasive prenatal testing (NIPT) is widely used in the screening of common fetal chromosome aneuploidy [2].

Conventional karyotyping provides an overview of the entire genome and can identify structural and numerical chromosome abnormalities. Chromosomal microarray analysis (CMA) is a method using array technology to detect chromosome abnormalities spanning less than 5 Mb [3]. Because CMA does not require cell culture, samples which cannot be cultured by conventional karyotyping can be analyzed with CMA, and CMA offers faster testing result. However, conventional karyotyping is limited to detect the rearrangement with a length longer than 5 Mb, which can be detected by CMA [4] and CMA cannot detect balanced translocations, which can be detected by conventional karyotyping [5].

Here we report the prenatal diagnosis and genetic counseling of a Xq24q25 deletion in a Chinese family with normal phenotype using NIPT, chromosomal GTG-banding and CMA.

## Methods

### Patients and samples

In 2019, a 28-year-old, gravida 1, para 0, woman underwent amniocentesis at 17 weeks of gestation because the noninvasive prenatal testing (NIPT) results revealed 9.8 Mb deletion from Xq24 to Xq25. Her husband was 27-year old. There was no family history of birth defects or genetic diseases. GTG-banding karyotype analysis was performed on cultured amniocytes and parental blood samples. CMA on uncultured amniocytes was performed using the Affymetrix CytoScan 750 K chip, which includes 550 k non-polymorphic markers and 200 k SNP markers.

## Results

**Fig. 1:**
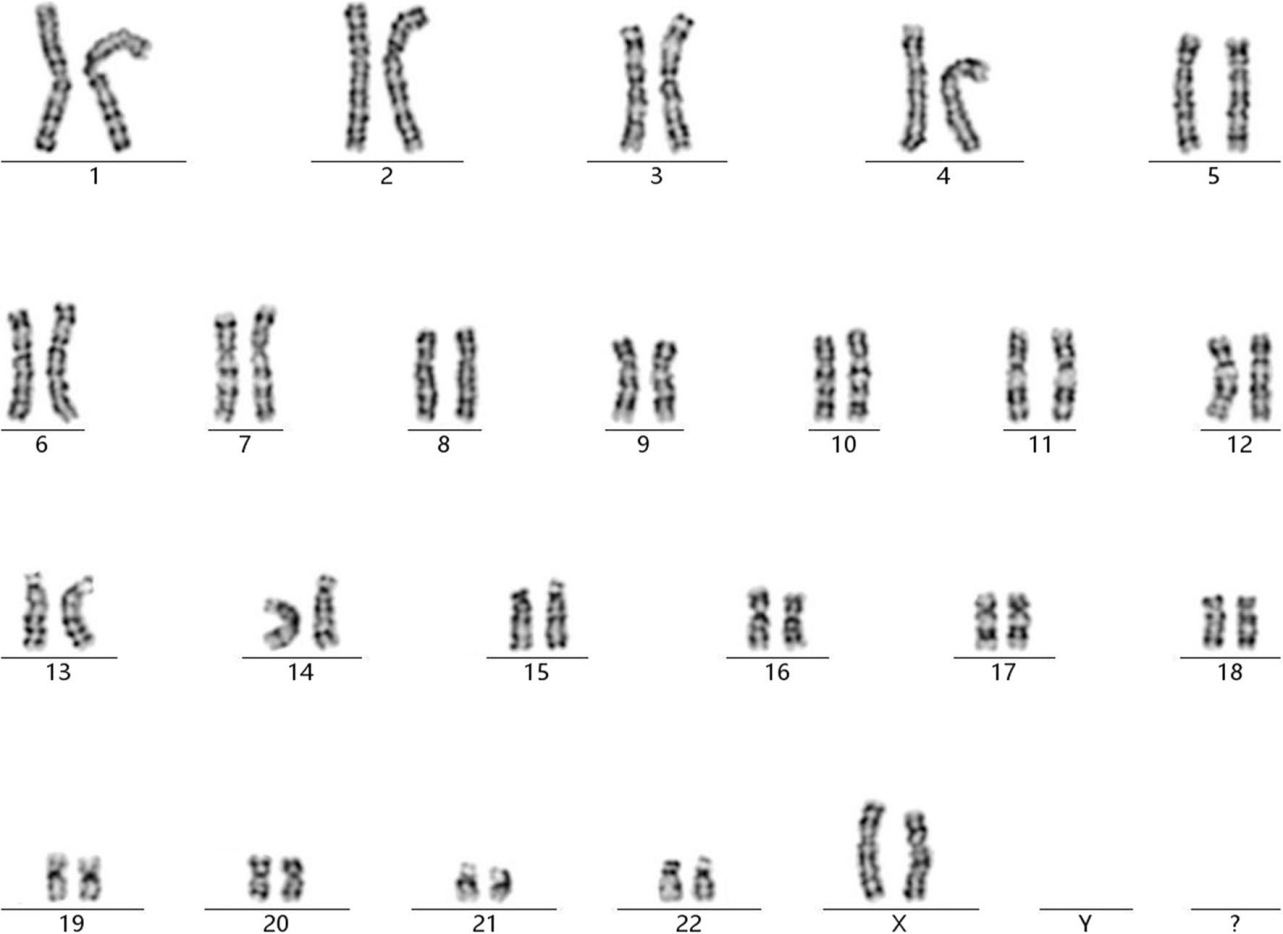
The karyotype of 46,XX

**Fig. 2:**
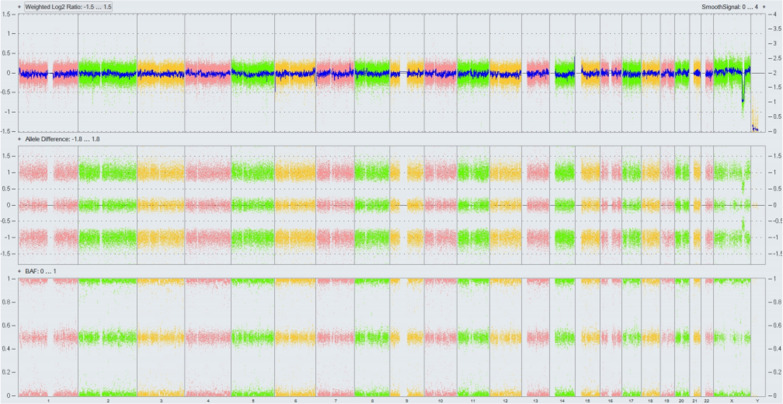
CMA detected a 9.8-Mb chromosomal deletion in the region of Xq24q25 (arr[GRCh37]Xq24q25(118,975,436_128,444,692) × 1)

Chromosomal GTG-banding revealed a karyotype of 46,XX (Fig. 1). CMA detected a 9.5-Mb chromosomal deletion in the region of Xq24q25, which is to be reported according to International System of Cytogenomic Nomenclature 2020 (ISCN 2020) [6] as arr[GRCh37] Xq24q25(118,975,436_128,444,692) × 1 (Fig. 2). Then we performed both CMA and chromosomal GTG-banding using the samples from the parents’ peripheral blood. Parental karyotypes were done and were 46,XX and 46,XY, respectively. However, in CMA the mother had the same deletion in Xq24q25 as the fetus. Ultrasound examination showed no dysmorphisms or intrauterine growth restriction (IUGR) in the fetus. A comprehensive physical examination of the parents, especially the mother showed no abnormalities.After genetic counseling, the parents decided to continue the pregnancy.

At 40 weeks of gestation, the expectant mother gave birth vaginally to a female baby. The baby’s growth parameters at birth were in the normal ranges. Apgar scores were 9/9/10. The baby received a complete physical examination and the results were normal. At 36-month checkup, the baby was developing normally (Intelligence Quotient, IQ = 108).

## Discussion

Only a few cases/families with Xq24q25 deletion have been reported in the literatures [1, 7–14]. The chromosomal deletion of Xq24q25 contains several dosage-sensitive genes, such as *LAMP2, CUL4B, XIAP, SH2D1A* and *GRIA3*. The deletion of *XIAP* and *SH2D1A* genes are the cause of X-linked lymphoproliferative disease [11]. The deletion of *LAMP2* gene is the cause of X-chromosomal dominant Danon disease [12, 13]. The deletion of *GRIA3* and *CUL4B* genes are the cause of X-linked mental retardation or/and X-linked intellectual disability [7, 12, 13].

NIPT is a very efficient and accurate method for the detection of chromosome aneuploidy, especially for chromosome 13, 18 and 21. Recently, further expansion of NIPT has focused on additional screening for sex chromosome aneuploidy. Maternal CNVs, especially at the X chromosome is an important cause of false positive NIPT results for sex chromosomal aneuploidy. In addition, some maternal CNVs can cause significant anomalies if the male fetus was inherited the X chromosome with CNVs [7].

X chromosomal CNVs does not usually cause signs or symptoms in women because of the presence of the second, normal X chromosome. Important genes in an X chromosome deletion CNVs can be recovered by the normal X chromosome [7]. However, if an X chromosome with a CNVs is transmitted to a male, it can cause a clinically significant phenotype.

Researches have shown that most female carriers with Xq24q25 deletion also develop symptoms, from a virtually asymptomatic to more classical profile. This variable expression in females is thought to be influenced by the process of X-chromosome inactivation (XCI). The analyses revealed that patients presented with the broad range of disease expression—from mild to severe, and their clinical involvement did not correlate with XCI profiles. Heterozygous female carriers with the random XCI may present with the wide range of disease signs and symptoms. Thus, XCI is not a main factor in the phenotype variability in heterozygous females [14, 15].

Generally in these cases the inactivated X is the one that is affected, with the deletion, i.e. there is a non-random inactivation of the X chromosome. But in a percentage of cases this non-random inactivation does not occur. Some authors suggest that this could be due to some type of gene or chromosomal aberration in the “normal X” [16].

Therefore, pregnant women with an X chromosomal CNVs need proper genetic counseling about the possible clinical outcomes. It is generally considered appropriate to offer genetic counseling about the potential risks to offspring and reproductive options to these female carriers [7].

During pregnancy, there were no dysmorphisms or IUGR in the female fetus. At the 3-year follow-up, the baby did not have an abnormal phenotype and exhibited no evidence of mental retardation, intellectual disabilit, X-linked lymphoproliferative disease or X-chromosomal dominant Danon disease. However, further study is needed. We plan to follow this patient in order to monitor her development.

CMA is superior to standard karyotype in detection of chromosomal microdeletion/microduplication [17]. Therefore, CMA is recommended as an additional diagnostic test while conventional prenatal tests including blood test, ultrasonography examination and invasive prenatal diagnosis revealed abnormal findings of fetus [17].

## Conclusions

Combination of NIPT, karyotype analysis, CMA, prenatal ultrasound and genetic counseling is helpful for the prenatal diagnosis of chromosomal microdeletions/microduplications.

## Data Availability

All relevant data and material is included in this publication.
